# Large outbreak of severe Shiga toxin-producing *E. coli* O157:H7 infections in multiple nursing homes in Belgium, August 2025

**DOI:** 10.1017/S0950268826101903

**Published:** 2026-07-29

**Authors:** Clara Mazagatos, Valeska Laisnez, Frederik De Keersmaeker, Aaron Devos, Gert Bellen, Britt Van Dijck, Catherine De Vocht, Naïma Hammami, Caroline Boulouffe, Eugénie Tchatchie, Tamara Colassin, David Hercot, Florence Rolin, Vera Cantaert, Anneline Christiaens, Koenraad Van Hoorde, Bavo Verhaegen, Bram Vanmechelen, Florence Crombé, Dieter Van Cauteren

**Affiliations:** 1 Department of Epidemiology and Public Health, Sciensano, Brussels, Belgium; 2 ECDC Fellowship Programme, Field Epidemiology path (EPIET), European Centre for Disease Prevention and Control (ECDC), Stockholm, Sweden; 3 Departement Zorg, Team Infection Prevention and Vaccination, Flemish Community, Brussels, Belgium; 4 Agence pour une Vie de Qualité (AVIQ), Charleroi, Belgium; 5 Vivalis, Brussels, Belgium; 6 Federal Agency for the Safety of the Food Chain, Brussels, Belgium; 7 National Reference Laboratory for Foodborne Outbreaks, Sciensano, Brussels, Belgium; 8 National Reference Centre for Shiga toxin-producing Escherichia coli, Universitair Ziekenhuis Brussel (UZ Brussel), Brussels, Belgium

**Keywords:** disease outbreaks, epidemiology, foodborne diseases, gastrointestinal diseases, Shiga-toxigenic *Escherichia coli*

## Abstract

Shiga toxin-producing *Escherichia coli* (STEC) O157 is a foodborne pathogen causing gastrointestinal illness. In August 2025, multiple nursing homes (NHs) in Belgium reported gastroenteritis cases among residents, which were later confirmed as STEC O157. A national outbreak investigation team was established to identify the source and implement control measures. Regional health authorities conducted field investigations, including NH visits, interviews, and exposure assessment. Microbiological and food chain investigations were coordinated nationally. We compared menus and initiated traceback investigations for high-risk food items for STEC. Using a retrospective case–control design, we calculated food-specific odds ratios and 95% confidence intervals. Additionally, we ranked exposures according to their permutation importance in a random forest prediction model. Investigations pointed to *filet américain*, consumed on 13 August, as the most plausible source. No leftovers were available for microbiological confirmation. We identified a batch of raw minced beef distributed to 104 institutions, including affected NHs, and found STEC-positive samples linked to it. With 71 cases and 10 deaths, this was among the largest STEC outbreaks in Belgium, prompting authorities to publish communications advising at-risk populations against the consumption of raw meat and underscoring the need to review existing recommendations on high-risk food items for foodborne infections.

## Introduction

Shiga toxin-producing *E. coli* (STEC) are zoonotic, foodborne pathogens capable of producing shiga toxins (*stx1* and *stx2*) and belonging to a wide range of serogroups. Ruminants, particularly cattle, are the main reservoir of STEC, where the bacteria colonize the gastrointestinal tract [1]. During slaughter and processing, faecal matter can contaminate food products with STEC. STEC infection in humans is most frequently associated with the consumption of undercooked minced beef, unpasteurized milk and dairy products, or raw fresh vegetables [2]. STEC serogroup O157:H7 has historically been linked to cattle and beef production chains, leading to large outbreaks associated with consumption of burgers and other ground beef products [3, 4].

STEC infections occur in the general population, but children and the elderly are at higher risk of severe disease outcomes. Symptoms range from mild diarrhoea to abdominal cramps, vomiting, and severe bloody diarrhoea [5]. A small proportion of STEC infections, particularly when shiga toxin genes *stx*_2a_ and *stx*_2d_ are present, can lead to haemolytic uremic syndrome (HUS) [6, 7], a complication characterized by haemolytic anaemia, thrombocytopenia, and acute renal failure. HUS is more frequent in young children, and it can lead to chronic renal disease and death (0.5–11% mortality) [6, 8–10].

Because of its potential for causing severe clinical outcomes and the threat of cross-border foodborne outbreaks, STEC is considered a pathogen of significant public health concern [11, 12], and surveillance and reporting of STEC infections are mandatory in most European Union (EU) member states. In 2024, STEC was the third most commonly reported foodborne zoonotic disease in the EU/EEA, with 35.7% of reported cases hospitalized and a mortality of 0.3% [11]. STEC O157:H7 was the most frequently reported serogroup in 2024, both in Belgium and the EU/EEA [11, 13, 14]. Existing European food safety guidelines and national recommendations in Belgium to reduce the risk of STEC and other foodborne infections emphasize standard good hygiene practices among food handlers and consumers, including adequate cooking, hand hygiene, and avoiding cross-contamination from raw to cooked products [13, 15]. However, despite the fact that there are defined vulnerable groups at risk of serious complications, preventive guidance specifically tailored for high-risk institutional settings is lacking, such as recommendations to avoid products prone to STEC contamination in childcare facilities or long-term care facilities for the elderly.

STEC surveillance in Belgium relies on the mandatory notification of STEC cases by clinicians and laboratories to the three regional health authorities [16–18], and on the surveillance carried out by the National Reference Centre (NRC) for STEC (Brussels University Hospital) that receives clinical samples and isolates from clinical laboratories for diagnosis and subtyping. Additionally, a network of sentinel laboratories (Epilabo) reports, on a weekly basis, test results for 40 different pathogens including STEC [19]. At the national level, surveillance is coordinated by Sciensano. In Belgium, 620 STEC cases were reported in 2024, with an increasing trend observed since 2021, likely related to the expanded use of culture-independent diagnostic techniques that facilitate STEC detection [14].

On 22 August 2025, Departement Zorg (DZ), the regional health authority in Flanders, informed Sciensano about an outbreak of cases presenting with gastrointestinal symptoms among individuals living in two nursing homes (NHs) in Flanders. Three other NHs reported cases on 25 August 2025. The severity of the outbreak, with four deaths reported at that time, rapidly raised public health concerns. We established a national multi-agency outbreak investigation team (OIT), including representatives from the regional health authorities (DZ in Flanders, *Agence pour une Vie de Qualité* (AVIQ) in Wallonia, and Vivalis in Brussels), the Belgian Institute for Health (Sciensano), the Federal Agency for the Safety of the Food Chain (FASFC), the NRC for STEC, and the National Reference Laboratory (NRL) for Foodborne Outbreaks (Sciensano). The aim of the OIT was to identify the potential source of the STEC infection, implement appropriate control measures to protect residents in the NHs, and make public health recommendations to prevent future outbreaks.

## Methods

### Outbreak case definitions

Possible cases were defined as residents of any Belgian NH presenting with gastrointestinal symptoms from 13 August 2025. Possible cases living in a Belgian NH with at least one case confirmed by the NRC were defined as probable cases. Confirmed cases were defined as residents of a Belgian NH with STEC infection confirmed by the local clinical laboratory or the NRC.

### Epidemiological investigation

After notification of the outbreak, regional health authorities (DZ, AVIQ, and Vivalis) conducted visits to affected NHs. They collected information about menus served during the suspected exposure period (11 to 19 August 2025), based on the STEC incubation period (3–8 days) [20]. Menus were compared to identify common food items served in all affected facilities. Food items that are known to be at risk for STEC infection were further investigated by the FASFC. To determine whether other countries were observing an increase in STEC O157 cases and to assess possible cross-border spread, we issued an alert through EpiPulse (the European surveillance portal for infectious diseases, run by the European Centre for Disease Prevention and Control (ECDC)) on 28 August 2025.

### Case–control study

A case–control study was conducted in the two most affected NHs, both in Flanders. We included all probable and confirmed cases identified in these NHs and two to three controls per case, depending on feasibility. Controls were selected among residents in the same NH who did not report gastrointestinal symptoms during the period 13–30 August 2025. Age differences in this population of NH residents are minimal, and no matching was done by age or any other variable. Between 29 August and 3 September, DZ conducted face-to-face interviews with structured questionnaires among cases and controls, and collected data on age, symptoms, date of onset, and consumption of food items identified in the menus served from 11 to 19 August 2025. In principle, residents were interviewed themselves. If not possible or feasible (i.e., for residents with dementia, or those too frail to answer) or when recall was limited or not feasible, the information was complemented through interviews with staff members.

DZ transmitted the raw data to the epidemiology team at Sciensano, who consolidated and cleaned the data and conducted the statistical analyses. This required translating menus, categorizing dishes into individual food items, grouping food items into categories, and preparing a structured line list for statistical analysis. Before the analysis, we excluded six cases whose data were deemed insufficient or too unreliable to determine case–control status or food exposure status. We conducted all analyses with pooled NH data to increase power. For each food exposure, we calculated both crude odds ratios (ORs), corrected ORs (applying a 0.5 continuity correction to all cell counts in the contingency tables), and 95% confidence intervals (CIs). As a complementary approach, we used a random forest (RF) model to predict case–control status based on food exposures. We then ranked food items according to their permutation importance, i.e., the decrease in prediction accuracy when exposure status for that food item is randomly reshuffled. Exposures that strongly contribute to the outcome prediction will exhibit a large decrease in accuracy when permuted and rank higher, while exposures with small predictive power lead to smaller changes in accuracy and rank lower. Moreover, the model may rank a food item that many controls but few cases consumed as high because of its usefulness in identifying controls rather than cases. However, while RF permutation importance does not encode the direction of association like ORs, it is less susceptible to the problem of multiple comparisons. Results from OR and RF analyses were interpreted together to identify the most probable source of the STEC contamination, combining formal hypothesis testing using ORs with a predictive RF ranking approach more robust to multiple comparisons and complex interactions between food items. All analyses were performed in R (https://www.r-project.org/).

### Microbiology and genome sequencing

Stool samples from possible and probable cases were sent to local clinical laboratories for initial screening and, if positive, subsequently referred to the NRC, or sent directly to the NRC for STEC screening, isolation, and characterization, including ‘Big Five’ serotyping (O26, O103, O111, O145, and O157) and virulence typing [21]. At the NRC, IS629 typing was performed directly on all O157-positive isolates to identify those with identical IS629 profiles prior to WGS [22]. Once available, sequencing reads generated on the MGI instrument (DNBSEQ-T7, MGI Tech Co., Shenzhen, China) were uploaded to EnteroBase for cluster assignment [23]. A threshold of ≤5 allelic differences (HC5) was applied for cluster detection. Isolate-specific identifiers are provided in Supplementary Table S1.

### Food traceback investigations

After examining menus and identifying food items that were served in the affected NHs, the FASFC initiated traceback investigations for all food items considered at risk for STEC contamination. Food and environmental samples were tested for STEC by the NRL for foodborne outbreaks using the ISO/TS 13136:2012 for the detection of STEC and ISO 16654:2001 for the specific detection of *E. coli* O157.

## Results

### Descriptive epidemiology

**Figure 1.**
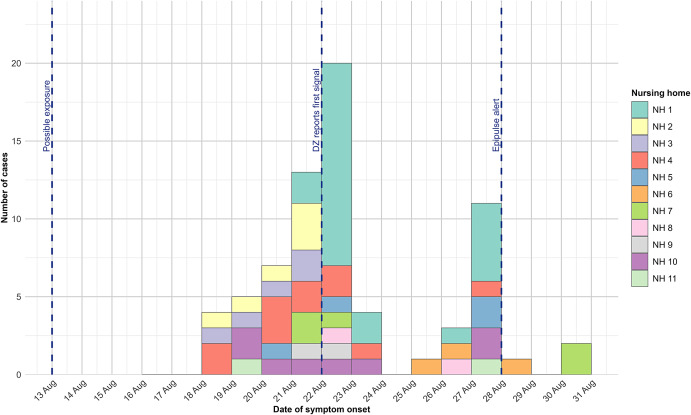
Epidemiological curve of probable and confirmed Shiga toxin-producing *E. coli* (STEC) cases by date of symptom onset and nursing home in Belgium, August 2025 (*n* = 71). ChatGPT (version GPT-5) was used to refine the R code that generated this figure.

Overall, the outbreak included 71 cases with symptom onset ranging from 18 to 30 August 2025 (Figure 1), among which 43 were probable cases, and 28 were laboratory confirmed as STEC (Table 1). Among confirmed cases, 20 were confirmed by the NRC as STEC O157:H7 *stx*_1a_^+^ *stx*_2a_^+^ *eae* ^+^ (HC5_357459). Confirmed cases in NHs were reported across all regions in Belgium, with nine NHs affected in Flanders, one in Brussels, and one in Wallonia (Table 1). Two cases with HUS were reported. The NRC identified one additional isolate belonging to the same cluster, from a symptomatic patient with no epidemiological link to the NHs (Supplementary Table S1). Eleven countries responded to our EpiPulse alert, and none of them reported cases matching the same genomic cluster.

**Table 1.** Number of probable cases, confirmed cases, and attack rates, overall and by nursing home, Belgium, August 2025 Table 1. long description.

Region	Affected NH	Probable cases	Confirmed cases	Probable and confirmed	Total NH residents	Attack rate (%)
Flanders	NH 1	19	4	23	146	15.8
	NH 2	5	1	6	90	6.7
	NH 3	1	4	5	125	4.0
	NH 4	7	4	11	172	6.4
	NH 5	1	3	4	90	4.4
	NH 6	0	3	3	72	4.2
	NH 7	3	2	5	90	5.6
	NH 8	1	1	2	97	2.1
	NH 9	0	2	2	80	2.5
Wallonia	NH 10	6	2	8	147	5.4
Brussels-Capital	NH 11	0	2	2	86	2.3
Total		43	28	71	1,317	5.4

NH, nursing home.

The overall attack rate among NH residents was 5.4% (71/1317), with high variability by NH. The highest attack rates were observed in three Flemish NHs: NH 1 (15.8%), NH2 (6.7%), and NH 4 (6.4%) (Table 1). In total, 10 residents died, corresponding to a case fatality of 14.1% (10/71). The time distribution of probable and confirmed cases shows a first peak of cases on 22 August and a second smaller peak on 27 August (Figure 1).

### Analytical epidemiology: Case–control study

In the case–control study, we included 19 cases and 67 controls from two NHs (NHs 1 and 4). The median age was 89 years (interquartile range (IQR): 81–93) among cases and 90 years (IQR: 83–93) among controls. Six cases (32%) reported bloody diarrhoea, while 13 (68%) had mild gastrointestinal symptoms, such as loose stool or watery diarrhoea without blood.

Results from the OR calculation and the RF permutation importance ranking are presented in Table 2. In the OR analysis, four food items were significantly associated with being a case: spinach soup (OR: 19.3, 95% CI: 1.1–334.2), warm vegetable mix (OR: 6.8, 95% CI: 1.6–28.9), ham (OR: 4.4, 95% CI: 1.1–18.1), and raw minced beef meat (OR: 3.8, 95% CI: 1.1–13.3). Warm vegetable mix and soups were not considered as high-risk for STEC (because they were cooked and served warm) or not offered in all affected NHs. Ham, which included cooked salted products from multiple brands and suppliers, was treated as a grouped exposure and is not typically associated with STEC contamination. Raw minced beef meat was considered the most likely source, as it is a known high-risk food item and was consistently served across affected facilities. In the RF analysis, raw minced beef was ranked as the sixth most important outcome predictor (Table 2).

**Table 2.** Number of exposed and unexposed cases and controls, and results of the odds ratio calculations and random forest ranking results for the top-ranked food items according to both methods Table 2. long description.

Food item	Exposed		Unexposed		Crude OR	Corrected OR		RF permutation importance ranking
	Cases	Controls	Cases	Controls		OR	95% CI	
Top 6 according to OR calculation								
Spinach soup	19	45	0	22	Inf	19.3	1.1–334.2	8
Celery soup	19	50	0	17	Inf	13.5	0.8–235.8	17
Carrot soup	19	51	0	16	Inf	12.5	0.7–218.5	25
Warm vegetable mix	6	5	5	31	7.4	6.8	1.6–28.9	5
Ham	16	38	2	25	5.3	4.4	1.1–18.1	2
Raw minced meat	16	36	3	29	4.3	3.8	1.1–13.3	6
Top 6 according to RF								
Sandwiches	7	27	5	9	0.5	0.5	0.1–1.8	1
Ham	16	38	2	25	5.3	4.4	1.1–18.1	2
Tomato mozzarella	1	12	5	12	0.2	0.3	0.04–2.0	3
Cheese	2	19	4	7	0.2	0.2	0.04–1.2	4
Warm vegetable mix	6	5	5	31	7.4	6.8	1.6–28.9	5
Raw minced meat	16	36	3	29	4.3	3.8	1.1–13.3	6

CI, Confidence interval; OR, Odds ratio; RF, Random Forest.

### Food investigations

We identified 100 food items served on the menus at the affected NHs. Three types of food items were considered high-risk for STEC and were further investigated by the FASFC: raw milk cheese, raw vegetables, and raw minced beef meat. A link was identified between the outbreak and a batch of raw minced beef from a Belgian producer that was distributed nationwide to 104 institutions, including all affected NHs. The suspected batch was served as *filet américain*, a traditional Belgian dish prepared from raw minced beef and consumed uncooked. It was included in the menus of all NHs on 13 August. No leftovers from the *filet américain* served in the NHs were available for microbiological testing. Other food samples were collected in the NHs and tested negative for STEC. Inspections were carried out at the facility that produced the suspected batch of minced meat. Traceback investigations identified bovine carcasses and frozen hamburgers that were sampled and tested positive for STEC although not for the O157 outbreak strain.

### Outbreak control measures

The last case had symptom onset on 30 August, and no additional cases were reported after that date. By the time our investigation identified *filet américain* as the most likely source of contamination, approximately 2 weeks after the exposure on 13 August, the outbreak was already over, and the suspected batch of raw minced meat had been consumed or discarded, so recall or withdrawal from the supply chain was not required.

During the outbreak, regional health authorities were in close communication with affected NHs and provided them with public health guidance, sharing information about STEC and measures to prevent further transmission among residents. Following the outbreak, the FASFC published general communications, advising people at risk to avoid consuming raw or undercooked meat products. A working group has been set up, involving all stakeholders at regional and national level, to update recommendations on food advice for residents in NHs and other vulnerable groups at risk for severe complications, such as young children, pregnant women and immunocompromised patients.

## Discussion

This study describes the investigation of a STEC O157:H7 outbreak that affected residents in 11 NHs across Belgium. By triangulating epidemiological, microbiological, and food chain investigations, our findings pointed to raw minced beef meat, served on 13 August 2025 in all affected NHs as *filet américain*, as the most plausible source of infection.

Raw minced beef meat is a known risk product for STEC O157 contamination. Previous outbreak investigations have reported links between STEC O157 infections and the consumption of undercooked beef burgers in France [3] and ground beef in the United States [4]. In this outbreak, the suspected batch of raw minced meat originated from a Belgian producer and was distributed nationwide to 104 institutions, including NHs, restaurants, hospitals, and other settings. However, only 11 NHs were affected by the outbreak, a relatively low number given the widespread distribution of the batch. This finding, together with the low overall attack rate among residents (5.4%) and the uneven impact across NHs, with one facility much more affected than the others, suggests that contamination of the suspected batch with STEC was low and heterogeneous. Through traceback investigations, we found samples from bovine carcasses and frozen hamburgers linked to the same batch that tested positive for STEC but negative for the O157 serogroup. Co-contamination of beef with O157 and non-O157 STEC strains is biologically plausible and has been observed in raw beef and carcass samples, reflecting the diversity of STEC serogroups carried by cattle [24].

According to the Belgian Health Care Knowledge Centre, the mean age of residents in homes for older people in Belgium has increased over time, reaching 87 years for women and 84 years for men in 2021 [25]. This reflects well the context of this outbreak, which affected a very old and frail population, and explains the high case fatality (14%) observed among NH residents. Despite the batch being distributed to a variety of institutions, there were no cases reported among young children, who are also at high risk of STEC, and only two cases presented HUS. One isolated community case, with no apparent connection to the NHs, was confirmed in a person who had consumed raw minced meat. This pattern suggests that a partial contamination of the batch of raw minced meat may have disproportionately affected the elderly residents of NHs due to a combination of weaker immune systems, comorbidities, and greater susceptibility to severe disease, along with dietary exposure to *filet américain*, a traditional speciality popular in the Belgian adult population. No other countries reported an increase in STEC O157 infections linked to the Belgian NHs.

The temporal distribution of cases suggests a point-source exposure, with a first peak on 22 August that is consistent with foodborne transmission following consumption of the *filet américain* served on 13 August 2025. The second smaller peak of cases on 27 August likely reflects secondary human-to-human transmission within the affected NHs. These findings provided a strong first hypothesis of the possible source, which was later supported by the analytical study results and the food chain investigations.

The consumption of raw meat products poses a known risk of foodborne outbreaks, especially in vulnerable populations. However, it remains a common practice in Belgium and other European countries such as Germany, where traditional raw pork products have been associated with recurrent outbreaks [26–28]. In 2013, a *Salmonella* outbreak in NHs in Germany was linked to traditional raw pork sausages, which were served in affected facilities despite national recommendations advising against it [29]. Similar to *filet américain* in Belgium, these raw pork sausages can be eaten as a spread on bread and are a very popular traditional food in some regions of the country. Interestingly, one reported reason for serving these products in the German NHs was that many elderly people strongly requested them, and even when they were not served by the facility, investigators suspected that visitors would provide residents with these products [29]. To prevent future similar outbreaks, NHs in Belgium could benefit from implementing risk mitigation measures, including staff training on food safety protocols, addressing the importance of the cold chain, and avoiding cross-contamination, particularly when serving high-risk products. When these products are offered, institutions should always ensure robust communication of associated risks to residents and provide safer alternatives. Enjoying tasty food can contribute to a sense of well-being in the final stages of life, and by providing residents with clear information and offering lower-risk alternatives, facilities can enable them to make informed and dignified choices in this regard. For residents unable to make informed decisions, facilities should involve family members in meal choices, ensuring that risks are clearly communicated and individual preferences are respected.

Raw meat is a perishable product with a short shelf life, so it is expected that the contaminated beef batch was either consumed or discarded in the days following delivery. There were no leftovers of *filet américain* available in the NHs. In this context, with absence of microbiological confirmation from food samples, our investigation relied entirely on epidemiological and food traceability evidence to identify the most probable source of the outbreak. In foodborne outbreak investigations, traceability of high-risk foods and epidemiological investigations remain essential approaches, not only when timely testing of perishable products is not feasible, but also when detection of the pathogen is difficult, particularly when contamination of the source is partial and heterogeneous. In the current era of widespread whole-genome sequencing, a perfect genomic match between human and food isolates is not always possible and, while providing very useful information, should not be an essential requirement for taking public health action. Epidemiological, traceback, and environmental investigations can often provide sufficient evidence to guide timely action, preventing additional cases, even in the absence of a definitive microbiological link – as this investigation shows. By using two analytical approaches, we combined traditional OR calculation for hypothesis testing with an RF machine-learning model for outcome prediction, which is more robust to multiple comparisons and complex interactions between food items. In both analyses, raw minced meat was among the highest-ranked items, strengthening our initial hypothesis. These results, combined with the traceback findings, allowed us to identify *filet américain* as the most probable source of the STEC outbreak in NHs.

We encountered several limitations while conducting the case–control study in the NH setting. First, the catering in these facilities included largely fixed menus, offering residents limited choice and resulting in a substantial overlap of food exposures between cases and controls. We had access to the weekly menus, but there was no prior record of specific food items included in the meals consumed by the residents. This made interviews and subsequent data management and food categorization quite challenging and time-consuming. Also, it might have caused exposure misclassification in both cases and controls, potentially influencing the OR estimate for raw minced meat. Second, interviews were conducted from 29 August to 2 September, 2 weeks after the consumption of the suspected *filet américain.* Thus, responses might have been affected by recall bias, as well as cognitive limitations and other health issues that we observed in the residents. For this reason, there was a need to closely collaborate with caregivers and other members of the staff, who provided support during data collection. Overall, these issues complicated the assessment of case status and food exposures in cases and controls, leading to the exclusion of some individuals from the OR and RF analyses. We initially included in the study one additional NH in Wallonia, where 7 cases and 6 controls were interviewed. However, the collected data from this NH was not granular enough to be useful and was eventually excluded from the analyses.

## Conclusions

This was among the most severe STEC outbreaks reported in Belgium, receiving intense national media attention due to its high case fatality and widespread impact across all regions. During the investigation, the priority was to identify as soon as possible the NHs that were affected, which required rapid confirmation by the NRC of at least one STEC O157 case per NH. By the time we identified raw minced meat as the most likely source of the STEC O157 infections, the outbreak was over. The suspected batch of raw minced beef meat had already been consumed or removed, so there was no need for recall or withdrawal actions. Following the outbreak, the FASFC issued communications advising people at risk of severe foodborne infections (the elderly, young children, pregnant women, and immunocompromised people) to avoid consuming raw or undercooked meat products. *Filet américain* is a traditional dish in Belgian cuisine and is often regarded as a delicacy. In this context, risk management recommendations need to balance food safety considerations with the quality of life of individuals living in institutionalized settings, including cultural and social factors. This balance should be addressed through targeted communication and multidisciplinary working groups. In Belgium, this outbreak highlighted the need to review existing practices, leading to the establishment of a working group involving all relevant stakeholders to develop recommendations on high-risk food items for foodborne infections.

## Supporting information

10.1017/S0950268826101903.sm001 Mazagatos et al. supplementary material Mazagatos et al. supplementary material

## Data Availability

Data are available upon reasonable request by email to the Food and Waterborne Diseases Unit in Sciensano (FWD@sciensano.be).

## Supplementary material

The supplementary material for this article can be found at http://doi.org/10.1017/S0950268826101903.

## Long descriptions

### Table 1. Long description

The table consists of 7 columns: Region, Affected N H, Probable cases, Confirmed cases, Probable and confirmed, Total N H residents, and Attack rate (%).

* Flanders Region:

- N H 1: 19 probable, 4 confirmed, 23 total cases, 146 residents, 15.8% attack rate.

- N H 2: 5 probable, 1 confirmed, 6 total cases, 90 residents, 6.7% attack rate.

- N H 3: 1 probable, 4 confirmed, 5 total cases, 125 residents, 4.0% attack rate.

- N H 4: 7 probable, 4 confirmed, 11 total cases, 172 residents, 6.4% attack rate.

- N H 5: 1 probable, 3 confirmed, 4 total cases, 90 residents, 4.4% attack rate.

- N H 6: 0 probable, 3 confirmed, 3 total cases, 72 residents, 4.2% attack rate.

- N H 7: 3 probable, 2 confirmed, 5 total cases, 90 residents, 5.6% attack rate.

- N H 8: 1 probable, 1 confirmed, 2 total cases, 97 residents, 2.1% attack rate.

- N H 9: 0 probable, 2 confirmed, 2 total cases, 80 residents, 2.5% attack rate.

* Wallonia Region:

- N H 10: 6 probable, 2 confirmed, 8 total cases, 147 residents, 5.4% attack rate.

* Brussels-Capital Region:

- N H 11: 0 probable, 2 confirmed, 2 total cases, 86 residents, 2.3% attack rate.

* Total:

- 43 probable cases, 28 confirmed cases, 71 total cases, 1,317 total residents, and an overall attack rate of 5.4%.

Navigate back to Table 1..

### Table 2. Long description

The table is organized into nine columns: Food item, Exposed Cases, Exposed Controls, Unexposed Cases, Unexposed Controls, Crude O R, Corrected O R, Corrected 95% C I, and R F permutation importance ranking.

Section 1: Top 6 according to O R calculation.

* Spinach soup: 19 exposed cases, 45 exposed controls, 0 unexposed cases, 22 unexposed controls. Crude O R is Inf, Corrected O R is 19.3, 95% C I 1.1–334.2, R F rank 8.

* Celery soup: 19 exposed cases, 50 exposed controls, 0 unexposed cases, 17 unexposed controls. Crude O R is Inf, Corrected O R is 13.5, 95% C I 0.8–235.8, R F rank 17.

* Carrot soup: 19 exposed cases, 51 exposed controls, 0 unexposed cases, 16 unexposed controls. Crude O R is Inf, Corrected O R is 12.5, 95% C I 0.7–218.5, R F rank 25.

* Warm vegetable mix: 6 exposed cases, 5 exposed controls, 5 unexposed cases, 31 unexposed controls. Crude O R is 7.4, Corrected O R is 6.8, 95% C I 1.6–28.9, R F rank 5.

* Ham: 16 exposed cases, 38 exposed controls, 2 unexposed cases, 25 unexposed controls. Crude O R is 5.3, Corrected O R is 4.4, 95% C I 1.1–18.1, R F rank 2.

* Raw minced meat: 16 exposed cases, 36 exposed controls, 3 unexposed cases, 29 unexposed controls. Crude O R is 4.3, Corrected O R is 3.8, 95% C I 1.1–13.3, R F rank 6.

Section 2: Top 6 according to R F.

* Sandwiches: 7 exposed cases, 27 exposed controls, 5 unexposed cases, 9 unexposed controls. Crude O R is 0.5, Corrected O R is 0.5, 95% C I 0.1–1.8, R F rank 1.

* Ham: (Data identical to Section 1), R F rank 2.

* Tomato mozzarella: 1 exposed case, 12 exposed controls, 5 unexposed cases, 12 unexposed controls. Crude O R is 0.2, Corrected O R is 0.3, 95% C I 0.04–2.0, R F rank 3.

* Cheese: 2 exposed cases, 19 exposed controls, 4 unexposed cases, 7 unexposed controls. Crude O R is 0.2, Corrected O R is 0.2, 95% C I 0.04–1.2, R F rank 4.

* Warm vegetable mix: (Data identical to Section 1), R F rank 5.

* Raw minced meat: (Data identical to Section 1), R F rank 6.

Navigate back to Table 2..
