# Supplementary material for: Large outbreak of severe Shiga toxin-producing E. coli O157:H7 infections in multiple nursing homes in Belgium, August 2025
Source: Epidemiol Infect. 2026 Jul 29;154:e106. doi: 10.1017/S0950268826101903 (PMC13437990; doi:10.1017/S0950268826101903)
Supplement: Supplementary file 1 [file S0950268826101903sup001.docx]

**Table S1**. Relevant microbial and patient information of all STEC O157:H7 isolates, with PubMLST ST11, included in the outbreak investigation.

| **Isolate ID** | **Stx1** | **Stx2** | **IS621 type** | **HC0 ^1^** | **HC5 ^2^** | **HC10** | | **Age** | **Clinical symptoms** | **Sampling date** | **NH of residence** | **Province** | **Accession** |
| --- | --- | --- | --- | --- | --- | --- | --- | --- | --- | --- | --- | --- | --- |
| EH4986 | 1a | 2a | AH | 357459 | 357459 | 30410 | | ≥65 | UNK | 2025-08-18 | NH 3 | Antwerp | ERR15536507 |
| EH4988 | 1a | 2a | AH | 357459 | 357459 | 30410 | | ≥65 | BD | 2025-08-21 | NH 9 | East Flanders | ERR15536520 |
| EH4989 | 1a | 2a | AH | 357459 | 357459 | 30410 | | ≥65 | BD | 2025-08-20 | NH 3 | Antwerp | ERR15536512 |
| EH4994 | 1a | 2a | AH | 357459 | 357459 | 30410 | | ≥65 | BD | 2025-08-20 | NH 4 | Flemish Brabant | ERR15536541 |
| EH4999 | 1a | 2a | AH | 357459 | 357459 | 30410 | | ≥65 | D | 2025-08-21 | NH 3 | Antwerp | ERR15536524 |
| EH5000 | 1a | 2a | AH | 357459 | 357459 | 30410 | | ≥65 | D | 2025-08-21 | NH 3 | Antwerp | ERR15536532 |
| EH5004 | 1a | 2a | AH | 357459 | 357459 | 30410 | | ≥65 | D | 2025-08-21 | NH 2 | Antwerp | ERR15536527 |
| EH5007 | 1a | 2a | AH | 357459 | 357459 | 30410 | | 15-24 | BD | 2025-08-23 | NA | West Flanders | ERR15536508 |
| EH5011 | 1a | 2a | AH | 357459 | 357459 | 30410 | | ≥65 | UNK | 2025-08-21 | NH 4 | Flemish Brabant | ERR15536516 |
| EH5013 | 1a | 2a | AH | 357459 | 357459 | 30410 | | ≥65 | UNK | 2025-08-21 | NH 4 | Flemish Brabant | ERR15536517 |
| EH5015 | 1a | 2a | AH | 357459 | 357459 | 30410 | | ≥65 | UNK | 2025-08-26 | NH 8 | East Flanders | ERR15536545 |
| EH5016 | 1a | 2a | AH | 357459 | 357459 | 30410 | | ≥65 | BD | 2025-08-26 | NH 6 | Flemish Brabant | ERR15536542 |
| EH5021 | 1a | 2a | AH | 357506 | 357459 | 30410 | | ≥65 | HUS | 2025-08-25 | NH 1 | Antwerp | ERR15536519 |
| EH5027 | 1a | 2a | AH | 357459 | 357459 | 30410 | | ≥65 | D | 2025-08-27 | NH 1 | Antwerp | ERR15652817 |
| EH5029 | 1a | 2a | AH | 357459 | 357459 | 30410 | | ≥65 | BD | 2025-08-27 | NH 10 | Walloon Brabant | ERR15652808 |
| EH5030 | 1a | 2a | AH | 357459 | 357459 | 30410 | | ≥65 | UNK | 2025-08-29 | NH 10 | Walloon Brabant | ERR15652820 |
| EH5032 | 1a | 2a | AH | 357459 | 357459 | 30410 | | ≥65 | D | 2025-09-01 | NH 11 | Brussels | ERR15652819 |
| EH5036 | 1a | 2a | AH | 357459 | 357459 | 30410 | | ≥65 | D | 2025-08-28 | NH 6 | Flemish Brabant | ERR15652810 |
| EH5037 | 1a | 2a | AH | 357459 | 357459 | 30410 | | 45-64 | D | 2025-09-03 | NH 7 | Flemish Brabant | ERR15652813 |
| EH5048 | 1a | 2a | AH | 357459 | 357459 | 30410 | | ≥65 | D | 2025-09-02 | NH 5 | Flemish Brabant | ERR15652818 |
| EH5055 | 1a | 2a | AH | 357459 | 357459 | 30410 | ≥65 | | D | 2025-09-09 | NH 1 | Antwerp | ERR15680865 |

^1^ HC0: hierarchical clusters (HC) with indistinguishable genomes; ^2^ HC5: hierarchical clusters (HC) with genomes up to 5 allele differences.

Clinical symptoms: BD: bloody diarrhoea; D: diarrhoea, HUS: haemolytic uraemic syndrome, NA: Not applicable, community case; UNK: unknown clinical symptoms, no HUS.
